# Delineating virulence of *Vibrio campbellii*: a predominant luminescent bacterial pathogen in Indian shrimp hatcheries

**DOI:** 10.1038/s41598-021-94961-4

**Published:** 2021-08-04

**Authors:** Sujeet Kumar, Chandra Bhushan Kumar, Vidya Rajendran, Nishawlini Abishaw, P. S. Shyne Anand, S. Kannapan, Viswas K. Nagaleekar, K. K. Vijayan, S. V. Alavandi

**Affiliations:** 1 ICAR-Central Institute of Brackishwater Aquaculture, 75, Santhome High Road, MRC Nagar, Chennai, 600 028 India; 2 ICAR - National Bureau of Fish Genetic Resources, Canal Ring Road, Dilkusha Marg, Lucknow, 226002 India; 3 ICAR -Indian Veterinary Research Institute, Izatnagar, Bareilly, 243122 India

**Keywords:** Microbiology, Molecular biology

## Abstract

Luminescent vibriosis is a major bacterial disease in shrimp hatcheries and causes up to 100% mortality in larval stages of penaeid shrimps. We investigated the virulence factors and genetic identity of 29 luminescent *Vibrio* isolates from Indian shrimp hatcheries and farms, which were earlier presumed as *Vibrio harveyi*. Haemolysin gene-based species-specific multiplex PCR and phylogenetic analysis of *rpoD* and *toxR* identified all the isolates as *V. campbellii*. The gene-specific PCR revealed the presence of virulence markers involved in quorum sensing (*luxM*, *luxS, cqsA*), motility (*flaA*, *lafA*), toxin (*hly, chiA*, serine protease, metalloprotease), and virulence regulators (*toxR, luxR*) in all the isolates. The deduced amino acid sequence analysis of virulence regulator ToxR suggested four variants, namely A123Q150 (AQ; 18.9%), P123Q150 (PQ; 54.1%), A123P150 (AP; 21.6%), and P123P150 (PP; 5.4% isolates) based on amino acid at 123rd (proline or alanine) and 150th (glutamine or proline) positions. A significantly higher level of the quorum-sensing signal, autoinducer-2 (AI-2, p = 2.2e−12), and significantly reduced protease activity (p = 1.6e−07) were recorded in AP variant, whereas an inverse trend was noticed in the Q150 variants AQ and PQ. The pathogenicity study in *Penaeus* (*Litopenaeus*) *vannamei* juveniles revealed that all the isolates of AQ were highly pathogenic with Cox proportional hazard ratio 15.1 to 32.4 compared to P150 variants; PP (5.4 to 6.3) or AP (7.3 to 14). The correlation matrix suggested that protease, a metalloprotease, was positively correlated with pathogenicity (p > 0.05) and negatively correlated (p < 0.05) with AI-2 and AI-1. The syntenic organization of *toxS-toxR-htpG* operon in *V. campbellii* was found to be similar to pathogenic *V. cholerae* suggesting a similar regulatory role. The present study emphasizes that *V. campbellii* is a predominant pathogen in Indian shrimp hatcheries, and ToxR plays a significant role as a virulence regulator in the quorum sensing—protease pathway. Further, the study suggests that the presence of glutamine at 150th position (Q150) in ToxR is crucial for the pathogenicity of *V. campbellii*.

## Introduction

Aquaculture is one of the fastest-growing food production sectors representing over 50% of world fish production. Along with capture fisheries, it supplies 17% of animal protein and supports the livelihoods of 12% of the world’s population^1^. One of the greatest threats to sustainable aquaculture is the existing and emerging pathogens. Vibriosis is a leading bacterial infection in marine and brackishwater farming systems^2^. The most serious problems are encountered in penaeid shrimp hatcheries, where luminescent vibriosis causes mortality up to 100% in mysis and early postlarval stages^3–5^. It also poses a serious threat to several cultured marine and brackish water finfishes such as Asian seabass, cobia, groupers, etc^2,6^.

The majority of the vibriosis outbreaks in aquatic animals is caused by closely related *Vibrio* spp*.* grouped under Harveyi clade comprising *V. harveyi, V. campbellii, V. owensii, V. alginolyticus, V. parahaemolyticus, etc*^2,6^. Due to high genetic and phenotypic similarities, conventional biochemical tests and 16 S rRNA often fail to differentiate species within Harveyi clade^7^. The average nucleotide identity (ANI) of 16S rRNA is considered as the gold standard for species delineation^8^. A 97% ANI threshold of 16S rRNA corresponding to 70% DNA-DNA hybridization has been widely used for species delineation^9^. However, there are reports that species of Harveyi clade share above 99% identity in 16S rRNA gene sequences and found inadequate to differentiate closely related bacterial species such as *V. harveyi* and *V. campbellii*^10^. In contrast to the 16S rRNA, several protein-coding house-keeping genes have a higher phylogenetic resolution at the species level. The multi-locus sequence analysis (MLSA) based on house-keeping genes is increasingly being applied for accurate phylogenetic analysis and differentiating closely related bacterial species^11^. Several house-keeping genes such as *topA*, *pyrH*, *ftsZ*, *mreB*, *rpoD*, *rctB* and *toxR* were found to differentiate species of Harveyi clade^7,12,13^. Recently, whole genome sequencing (WGS) has also been successfully employed for accurate identification of the bacterial pathogens^14,15^. Based upon whole genome sequence analysis, many of the *V. harveyi* strains have been reclassified as *V. campbellii*^16–18^.

The virulence of bacterial pathogens determines the severity and outcome of bacterial infections. Many virulence mechanisms such as quorum sensing, toxins production, type III secretion system, lysogenic phages, biofilm formation, haemolysin, proteases, chitinase, iron sequestering siderophore molecules, etc. have been reported to play a crucial role in the pathogenesis of *Vibrio* spp.^19,20^. Among the various virulence factors, protease activity was reported to have a significant role in the virulence of *Vibrio* spp*.* For example, it is reported that luminescent bacterium *V. harveyi* secretes cysteine protease^21^ and serine protease^22^ in a strain-dependent manner and is toxic to aquatic animals. Likewise, quorum sensing (QS) is the most characterized virulence regulatory mechanism in *V. harveyi*. It is a bacterial communication process that involves the secretion and detection of extracellular signaling molecules called autoinducers (AIs). Quorum sensing is known to controls bioluminescence^23^, siderophore, and exopolysaccharide production^24^ that negatively regulates Type III secretion system^23^ and upregulates the production of metalloprotease^25^ in a population density-dependent manner. Apart from quorum sensing, *toxR* is an important trans-membrane virulence regulator widely present in *Vibrio* species^26^. It is known to regulate the expression of virulence genes such as *ctxAB* (cholera toxin), *tcpA* (toxin-coregulated pilus, a colonization factor) in *V. cholerae*^26^*,* and expression of type VI secretion system 1 (T6SS1) in *V. parahaemolyticus*^27^. Although the role of *toxR* in pathogenicity has been reported in human pathogens such as *V. cholerae, V. parahaemolyticus,* its role in aquatic luminescent *Vibrio* spp. remains unknown. Moreover, there is a dearth of scientific data on the mechanism behind the avirulent and virulent strain of luminescent vibrios.

For a long time, *V. campbellii* was considered a non-pathogenic species. However, emerging reports of its frequent misidentification with *V. harveyi* in the past^16,18,28^ and its association with AHPND^29,30^ have enthused the focus to characterize the virulence behaviour of this pathogen. However, our understanding of the pathogenicity of *V. campbellii* is mainly limited to quorum sensing study on *V. campbellii* BAA-1116^23^, and the virulence mechanism of the species largely remains unknown. Against this background, the present study was envisaged to characterize the luminescent *Vibrio* isolates collected from the Indian shrimp hatcheries and farms (2006–2018) using 16S rRNA, *rpoD,* and *toxR*. The virulence mechanism was delineated by assessing the role of virulence markers, its association with allelic differences in *toxR*, and its pathogenic potential in *Penaeus* (*Litopenaeus*) *vannamei* juveniles.

## Results

The isolates LB515 and LB516 were isolated from *P. vannamei* postlarvae, while the remaining LB isolates were recovered from *P. monodon* hatcheries or farms (Table 1). The isolates were recovered from seven different locations, and 76% of isolates were collected from the hatchery set-up.

**Table 1:** *Vibrio* isolates used for the study.

Sl. no	Isolate no	Details of isolation	Luminescent disease affected hatchery	Year	Location
1	LB1	Brooder gills	Yes	2006	Marakanam, TN
2	LB 3	Spawning tank water	Yes	2006	Marakanam, TN
3	LB 10	Brooder body surface	Yes	2006	Marakanam, TN
4	LB 14	Broodstock maturation tank water	Yes	2006	Marakanam, TN
5	LB 16	Brooder intestine	No	2006	Marakanam, TN
6	LB 25	Faecal matter	No	2006	Marakanam, TN
7	LB 27	Brooder surface	No	2006	Marakanam, TN
8	LB 33	Brooder surface	No	2006	Marakanam, TN
9	LB 37	Broodstock maturation tank water	Yes	2006	Marakanam, TN
10	LB 39	Brooder surface	Yes	2006	Marakanam, TN
11	LB 67	Faecal matter	No	2006	Marakanam, TN
12	LB 102	Broodstock maturation tank water	Yes	2006	Marakanam, TN
13	LB 131	Pond water	No	2007	Mamallapuram, TN
14	LB 135	Pond sediment	No	2007	Nagapatinam,TN
15	LB 157	Pond water	No	2007	Nagapatinam,TN
16	LB 164	Pond water	No	2007	Nagapatinam,TN
17	LB 171	Pond water	No	2007	Ongole, AP
18	LB 178	Pond sediment	No	2007	Ongole, AP
19	LB 186	Shrimp gills	No	2007	Ongole, AP
20	LB 195	Postlarvae	Yes	2007	Marakanam, TN
21	LB 198	Postlarvae	Yes	2007	Marakanam, TN
22	LB 204	Postlarvae	Yes	2007	Marakanam, TN
23	LB 210	Postlarvae	Yes	2007	Kakinada, AP
24	LB 217	Postlarvae	Yes	2007	Marakanam, TN
25	LB 235	Mysis	Yes	2007	Marakanam, TN
26	LB 314	Brooder body surface	Yes	2008	Nagapatinam,TN
27	LB 503	Postlarvae	Yes	2009	Kakinada, AP
28	LB515	Postlarvae	Yes	2018	Visakhapatnam, AP
29	LB516	Postlarvae	Yes	2018	Chennai, TN
30	*V. campbellii* BAA- 1116	Seawater	No	1993	ATCC

*AP* Andhra Pradesh; *TN* Tamil Nadu; *ATCC* American Type Culture Collection.

### Phenotypic characterization

The biochemical profiles of 29 *Vibrio* isolates and one reference strain are presented in Supplementary Figure S1. All the isolates produced green colony on TCBS agar, grown in media supplemented with 3–6% sodium chloride, and were Gram-negative. This indicated that all the isolates were halophilic *Vibrio* species. However, none of the isolates recorded growth at 0% and 10% salt concentration. All the isolates were positive for lysine decarboxylase and fermentation of glucose, mannitol, cellobiose, galactose, mannose, and consistently negative for arginine dihydrolase, Voges-Proskauer reaction, and fermentation of sucrose, arabinose, and sorbitol. However, the ornithine decarboxylase test was positive for 86.2% of the isolates.

### Identification of *Vibrio* isolates by multiplex PCR

The species-specific multiplex PCR using the haemolysin gene of 29 *Vibrio* isolates and reference strain *V. campbellii* BAA1116 produced 328 bp PCR product specific for *V. campbellii* (Supplementary Figure S2).

### Identification of *Vibrio* isolates based on 16S rRNA, *rpoD* and *toxR* genes

The partial sequence of 16S rRNA, *rpoD*, and *toxR* genes were used for the identification of the isolates in the present study. The 16S rRNA had an average of 99.87%, 99.30%, and 98.99% identity with type strain *V. campbellii* CAIM519T, *V. owensii* CAIM 1854T, and *V. harveyi* ATCC 14126, respectively (Supplementary Table S2). It was far greater than the 97% identity threshold to categorize the isolates as different species. The *rpoD* had a maximum identity with *V. campbellii* CAIM 519T (average 99.24% identity) followed by *V. owensii* CAIM 1854T (96.14%) and *V. harveyi* ATCC 14126 (average identity 95.90%). The *toxR* had an excellent species differentiation ability with above 94% identity with *V. campbellii* CAIM519T compared to 82.94% with *V. owensii* CAIM 1854T and 75.00% average identity with *V. harveyi* ATCC 14126 (Supplementary Table S2).

**Figure 1:**
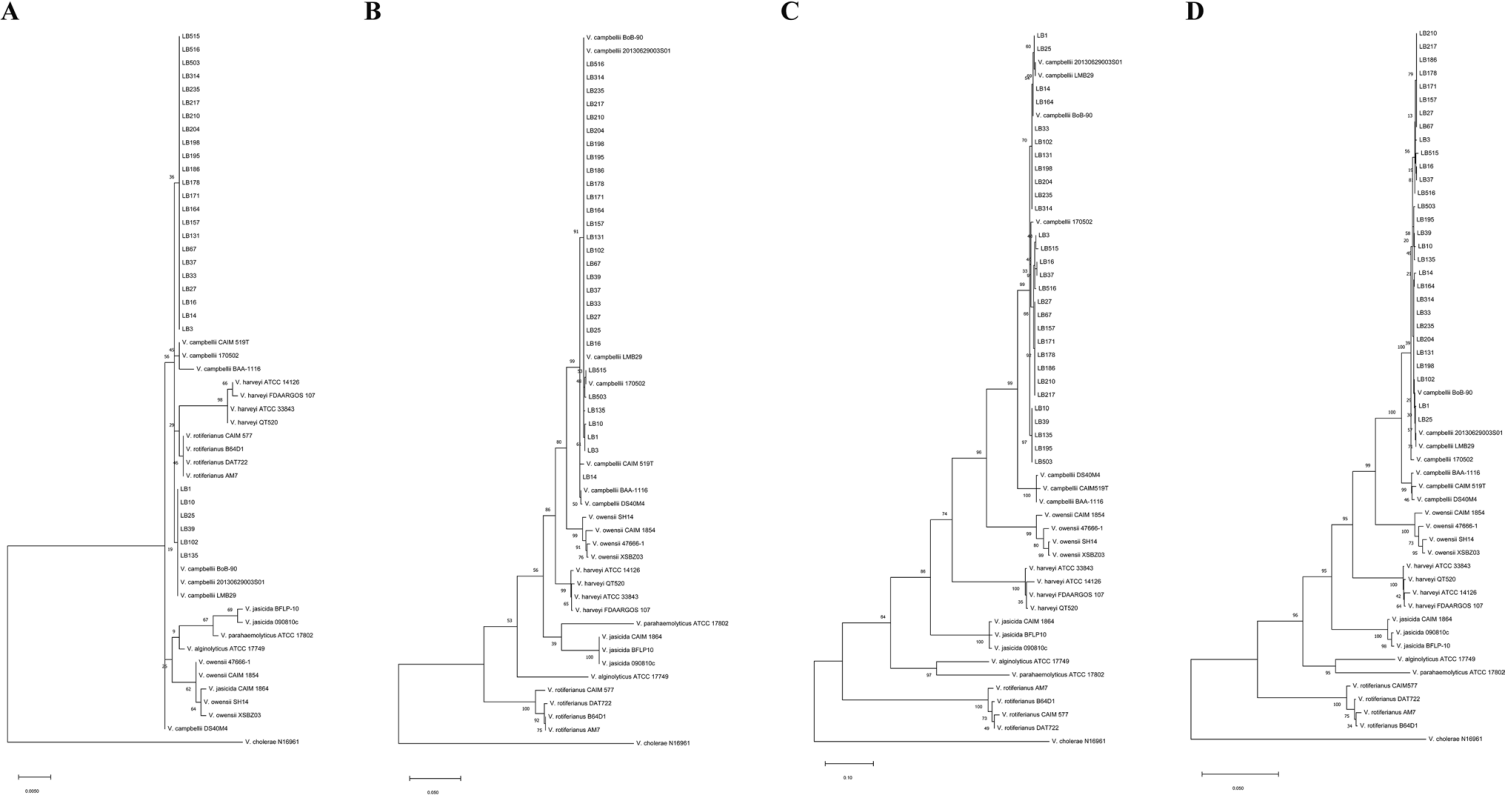
Maximum likelihood phylogenetic tree of luminescent bacterial isolates and other species under Harveyi clade was constructed using (**A**) 16S rRNA (**B**) *rpoD* (**C**) *toxR* and (**D**) concatenated sequence of 16S rRNA, *rpoD* and *toxR*. The tree was constructed at 1000 bootstraps replication and the length of each branch is proportional to the estimated number of substitutions per position. The tree is rooted using *V. cholerae* as outgroup. The 29 isolates of present study are represented by LB1 to LB516.

The maximum likelihood (ML) phylogenetic tree was constructed using 16S rRNA, *rpoD* and *toxR,* and their concatenated sequences (Figure 1). The amplicon length of 1402 nt for 16S, 807 nt for *rpoD* and 533 nt for *toxR* were used for constructing the phylogenetic tree. Contrary to the size, the largest number of parsimony-informative sites were for *toxR* (304 nt) followed by *rpoD* (169 nt) and the least for the 16S rRNA (35 nt). This was reflected in tree topology as 16S rRNA generated polyphyletic tree for *V. campbellii* and failed to differentiate the species of Harveyi clade. In comparison, *rpoD, toxR* and concatenated sequence of three genes identified all the isolates under study as *V. campbellii* and had monophyletic topology. The analysis further suggested that *V. campbellii* was phylogenetically most closely related to *V. owensii*, followed by *V. harveyi, V. jasicida, V. alginolyticus, V. parahaemolyticus,* and *V. rotiferianus*.

### Analysis of *ToxR* protein

**Figure 2:**
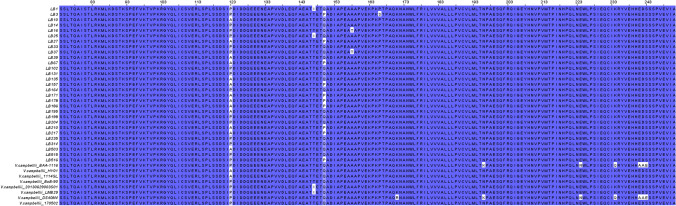
Amino acid sequence alignment of the variable region of ToxR of *V. campbellii.*

The full length ToxR protein in *V. campbellii* BAA-1116, *V. parahaemolyticus* RIMD2210633 and *V. cholerae* N16961 contain 290, 292 and 294 amino acids, respectively. The ToxR protein of *V. campbellii* BAA-1116 has 54.24% identity and 70% similarity with ToxR of *V. cholerae* N16961. In the present study, the amino acid sequence of the *V. campbellii* isolates was deduced from the partial nucleotide sequence of *toxR* gene. This resulted in amino acid composition between 74 and 250 AA which is 61% of the complete length. The multiple sequence alignment suggested a variable zone in the region of 120 to 170 AA of ToxR protein and is presented in Figure 2. The result suggests two critical amino acid substitutions at 123rd (proline to alanine) and 150th positions (glutamine to proline). This resulted from the change at 367th and 449th position of nucleotide leading to a change in codon from CCT to GCT (proline to alanine) and CAA to CCA (glutamine to proline). The deduced amino acid sequence of virulence regulator ToxR suggested four variants namely A123Q150 (AQ; 18.9%), P123Q150 (PQ; 54.1%), A123P150 (AP; 21.6%) and P123P150 (PP; 5.4% isolates).

**Figure 3:**
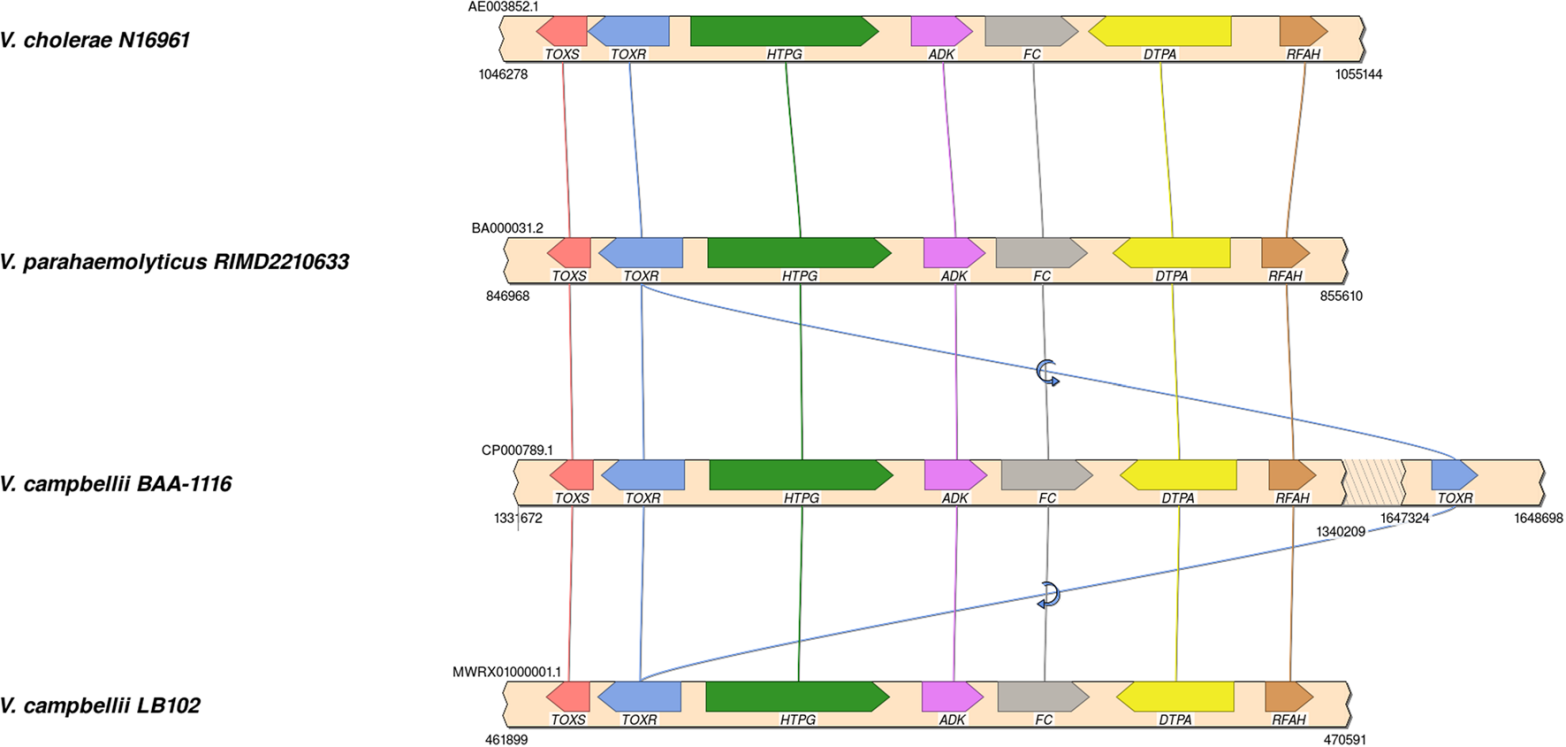
Syntenic gene organization of *toxR* gene loci in *V. campbellii, V. cholerae* and *V. parahaemolyticus.* HTPG: High Temperature Protein G (heat shock protein G); ADK: Adenylate kinase; FC: Ferrochelatase; DTPA: Dipeptide/tripeptide permease; RFAH: Transcription antitermination protein.

The syntenic organization of *toxR* gene loci is presented in Figure 3. The comparative analysis suggests that the syntenic organization of *toxR* gene loci in *V. campbellii* is similar to *V. cholerae* N16961 and *V. parahaemolyticus* RIMD2210633. These three *Vibrio* spp have *toxS* gene at the downstream of *toxR*. The heat shock protein *htpG* was found upstream and oriented in the opposite direction of the *toxRS* operon.

### Screening of virulence markers

**Figure 4:**
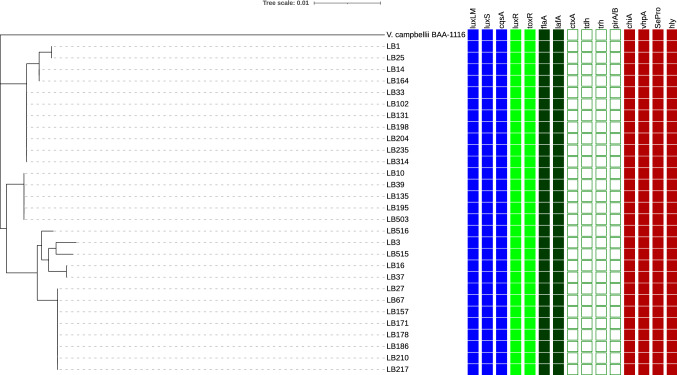
Distribution of virulence markers in *Vibrio campbellii* isolates based on gene-specific PCR. The phylogenetic tree was constructed based on *toxR* gene using maximum likelihood algorithm. The presence and absence of virulence markers was depicted by applying an additional panel using iTOL v4 (Interactive Tree of Life). The blue, red, green, and black shaded mark represents presence, while the white square box represents the absence of the gene.

Virulence markers present in the isolates under study are presented in Figure 4. All the 29 isolates possessed quorum sensing genes (*luxM*, *luxS, cqsA*), motility genes (*flaA* and *lafA*), toxin genes (*hly*, *chiA*, serine protease, metalloprotease), and virulence regulators (*toxR, luxR*). None of the tested isolates were positive for cholera toxin, *tdh*, *trh* and *pir* genes.

### Quantitative analysis of virulence markers

**Figure 5:**
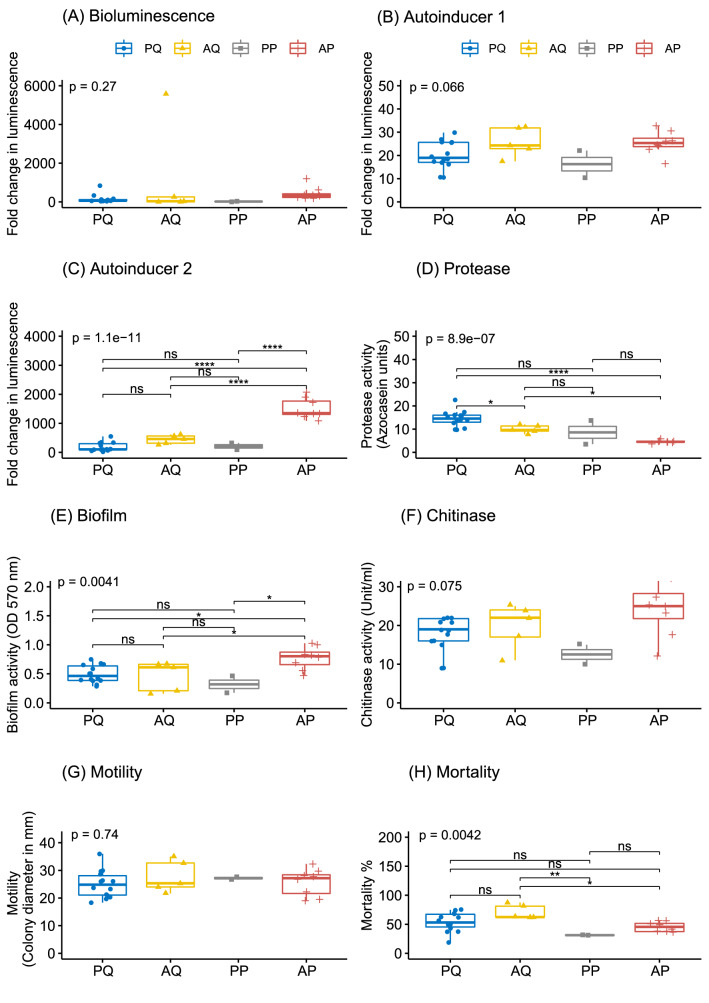
Box plot analysis of virulence markers and pathogenicity in *Vibrio campbellii* isolates among the variants of ToxR. (**A**) Luminescence, (**B**) Autoinducer 1 (AI-1), (**C**) Autoinducer 2 (AI-2), (**D**) Protease, (**E**) Biofilm, (**F**) Chitinase, (**G**) Motility (**H**) Mortality. One way ANOVA followed by Tukey post hoc comparison was carried out using rstatix package. The graphics were prepared using ggpubr package in R4.0.2. The AQ (A123Q150), PQ (P123Q150), AP (A123P150) and PP (P123P150) represents four variants of ToxR based upon amino acid at 123rd and 150th position.

In the present study, all the isolates were analysed quantitatively for luminescence, autoinducer signals AI-1, AI-2, protease, biofilm, chitinase, motility, and the result is presented in Figure 5. A significant difference was observed among the four allelic forms of ToxR for AI-2 (p = 1.1e−11), protease (p = 8.9e−07) and biofilm (p = 0.004) activity. Among the groups, glutamine to proline substituted AP group had the highest level of the autoinducer-2 signal (1506.0 ± 7.7) followed by AQ (443.5 ± 9.5), PP (205.4 ± 9.2), and PQ group (179.3 ± 16.1). In contrast, a reverse trend was observed for protease activity as the highest level was observed in PQ group (14.57 ± 0.72 U) and the lowest level in AP (4.62 ± 0.27 U). Similar to AI-2 signal, the highest biofilm activity was observed in AQ group.

There was no significant difference among the groups for quorum-sensing signal autoinducer-1 (p = 0.066), chitinase (p = 0.075) luminescence (p = 0.27), and motility (p = 0.74). Though the difference was insignificant (p = 0.08), the level of chitinase was the highest in the glutamine to proline substituted AP group. Except for LB3, all other isolates under study were found to be luminescent (Supplementary Figure S3). The pathogenicity of the *Vibrio* isolates did not find any significant correlation with luminescence. For example, pathogenic isolate LB503 was the most luminescent, while non-pathogenic isolates such as LB171 and LB186 also showed a higher level of luminescence. In contrast, many low luminescent isolates such as LB10, LB39, and LB195 were among the most pathogenic strains.

### Protease characterization

As shown in Table 2 the protease activity was completely inhibited by 1,10- phenanthroline and 60 to 100% inhibition by EDTA, indicating that the enzyme is metalloprotease. However, the activation of enzyme activity was observed with benzamide and PHMB, while PMSF had not much influence.

**Table 2:** Effect of inhibitors on protease activity in *Vibrio campbellii.*

Isolates	Protease	Protease activity in presence of inhibitors				
		EDTA (10 mM)	1,10-Phenanthroline (1 mM)	PMSF (1 mM)	PHMB (1 mM)	Benzamide (1 mM)
*V. campbellii* LB1	100	44	5	109	148	115
*V. campbellii* LB10	100	39	4	121	125	143
*V. campbellii* LB14	100	− 14	− 11	111	89	107
*V. campbellii* LB16	100	16	6	73	110	129
*V. campbellii* LB25	100	12	12	136	124	152
*V. campbellii* LB33	100	9	− 9	115	109	141
*V. campbellii* LB37	100	27	0	100	124	125
*V. campbellii* LB39	100	18	15	118	121	129
*V. campbellii* LB102	100	17	− 4	96	123	140
*V. campbellii* LB131	100	30	0	109	120	130
*V. campbellii* LB135	100	6	12	118	135	165
*V. campbellii* LB164	100	20	0	105	118	138
*V. campbellii* LB195	100	15	27	109	124	136
*V. campbellii* LB198	100	18	7	111	123	139
*V. campbellii* LB204	100	14	8	108	116	153
*V. campbellii* LB235	100	28	2	106	123	128
*V. campbellii* LB314	100	24	0	104	113	133
*V. campbellii* LB503	100	21	5	114	121	138
*V. campbellii* LB515	100	9	0	118	127	145
*V. campbellii* LB516	100	14	0	118	136	164
*V. campbellii* BAA-1116 BB120	100	35	8	115	138	154

The concentration of protease inhibitors applied are presented in parenthesis.

*EDTA* ethylenediaminetetraacetic acid; *PMSF* phenylmethylsulfonyl fluoride; *PHMB* p-hydroxymercuribenzoate.

The BLAST analysis of the EmpA of *V. anguillarum* against protein sequences of *V. campbellii* LB102 suggested the presence of three extracellular zinc metalloproteases of M4 peptidase family (Table 3). These metalloproteases shared 42.5 to 47.3% identity with EmpA of *V. anguillarum*. Further, one of these metalloprotease shared 96% identity with Pap6 metalloprotease/ hemagglutinin of *V. harveyi*. The BLAST analysis suggested the presence of extracellular collagenase of M9 family which shared 30.4% identity with collagenase of *V. alginolyticus*.

**Table 3:** Extracellular zinc metalloprotease in *Vibrio campbellii* LB102.

Gene description (species)	Accession No. of query sequence	Peptidase /domain family	*V. campbellii* LB102 protein accession no	BLAST score	Query coverage (%)	E value	% Identity
EmpA (zinc metalloprotease of *V. anguillarum*)	WP_194663493.1	M4	OPH49211.1	492	93%	1e−167	47.25%
		M4	OPH52411.1	493	98%	3e−167	44.61%
		M4	OPH49886.1	447	96%	2e−150	42.55%
Collagenase (*V. alginolyticus*)	AAZ06360.1	M9	OPH55752.1	265	66	8e−78	30.42%
Pap6 (Zinc metalloprotease/ hemagglutinin of *V. harveyi*)	AAM34261.1	M4	OPH49886.1	1313	100%	0.0	96.02%

### Principal component analysis

**Figure 6:**
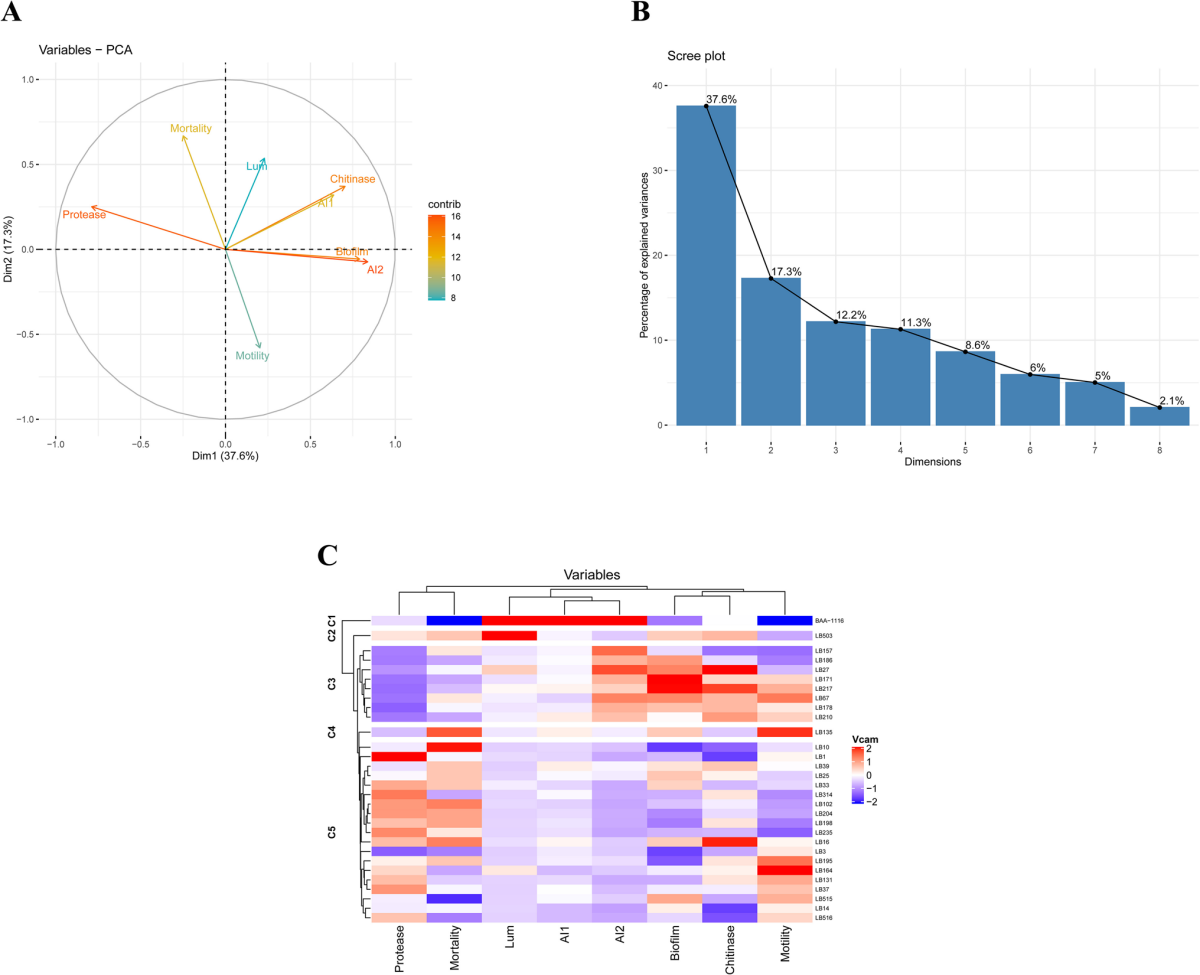
Principal component and cluster analysis of virulence factors and pathogenicity. (**A**) Principal component analysis. Dim1, First principal component; Dim2: second principal component; Lum: Luminescence; AI-1: Autoinducer-1; AI-2: autoinducer-2. (**B**) Scree plot showing the first three dimension for most of the cumulative variability in data represented by the eigenvalues (**C**) Hierarchical clustering analysis with the heatmap. The red to blue transition indicates a higher to a lower value.

For understanding the virulence, principal component analysis of quantitative virulence factors and pathogenicity data was carried out. The AI-1, AI-2, biofilm, chitinase, and protease were the major contributor in the first component and accounted for 37.6% of the total variance. Moreover, the direction of protease was opposite to AI-1, AI-2, biofilm, and chitinase. Mortality, motility, and luminescence were the major contributor in the second component and accounted for 17.3% of the total variance (Figure 6A). The observation that the first two principal components account for only 54.9% of the total variance indicates the heterogeneity within the virulence data (Figure 6B).

### Correlation matrix and cluster analysis

**Figure 7:**
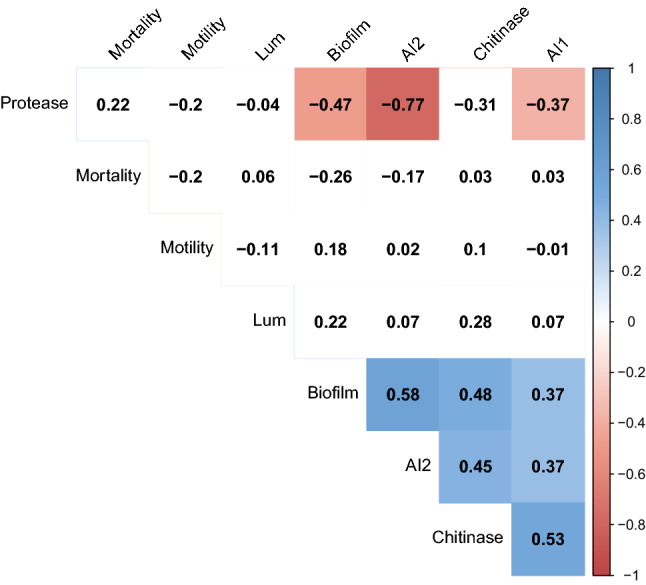
Correlation matrix (Pearson correlation coefficients) among virulence factors and pathogenicity. The highlighted block indicates a significant relationship (p < 0.05). Blue shade represents a significant positive correlation, while red indicates a significant negative correlation.

The correlation matrix among the virulence variables suggests that AI-1, AI-2, biofilm, and chitinase have a significant (p < 0.05) positive correlation (Figure 7). However, protease activity is negatively correlated (p < 0.05) with AI-1, AI-2, and biofilm formation activity and insignificantly positively correlated (p > 0.05) with mortality. The result was further supported by the heatmap analysis, which suggests that among the measured virulence factors, the pathogenicity of the isolates had the closest relation with protease activity (Figure 6C). However, the bioluminescence made a clustering pattern with quorum sensing signals AI-1 and AI-2.

### Pathogenicity analysis

**Figure 8:**
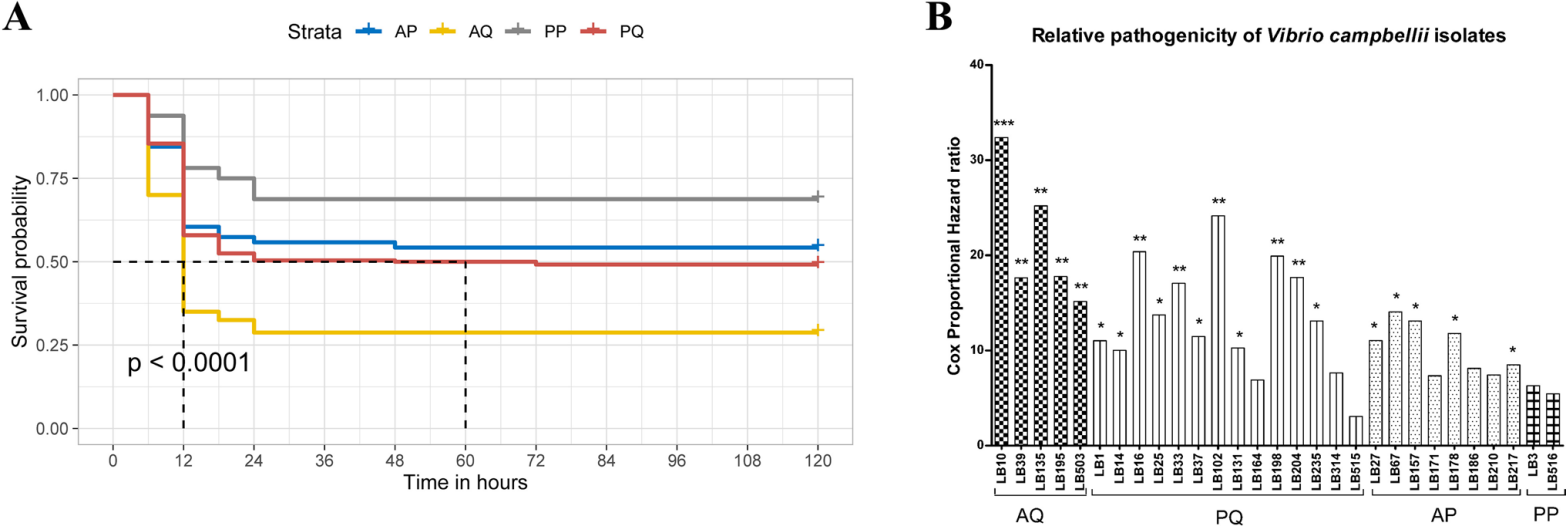
Pathogenicity of *Vibrio campbellii* in *Penaeus vannamei* juveniles. (**A**) Kaplan–Meier survival estimates of four variants of ToxR. (**B**) Relative pathogenicity of *V. campbellii* isolates against reference strain *V. campbellii* BAA-1116. The result was analysed by Cox proportional hazard analysis. The significance level is indicated at * (p < 0.05), ** (0.01) and *** (p < 0.001). The isolates within a ToxR variant is represented with a similar pattern. The AQ (A123Q150), PQ (P123Q150), AP (A123P150) and PP (P123P150) represents four variants of ToxR based upon amino acid at 123rd and 150th position.

The pathogenicity study was carried out in *P. vannamei* juveniles. The data was analysed by ANOVA (Figure 5H) and Kaplan–Meier method (Figure 8A) to find the survival difference among the ToxR allelic groups. ANOVA took the consideration of final survival data while Kaplan-Meir curve compared the survival among the groups at each time point. The pathogenicity of individual isolates was further compared against the least virulent reference strain *V. campbellii* BAA-1116 applying Cox proportional hazard analysis (Figure 8B). The analysis revealed a highly significant variation (p < 0.0001) in survival probability among the ToxR allelic groups (Figure 8A). The isolates with alanine at 123rd and glutamine at 150th position (group AQ) were the most virulent and had significantly higher level of pathogenicity compared to P150 variants AP (p < 0.05) and PP (p < 0.01) (Figure 5H). This was reflected in Cox proportional hazard ratio as it ranged between 15.1 to 32.4 for the isolates of AQ compared to only 5.4 to 6.3 for PP and 7.3 to 14 for AP (Figure 8B). Further, a higher level of pathogenicity was also recorded in PQ group compared to P150 variants PP and AP, though the difference was not significant. This was reflected by higher variation in the hazard ratio of PQ group which ranged between 3.1 and 24.2. Overall, 72.4% isolates had significantly higher (p < 0.05) pathogenicity with a hazard ratio 8.5 to 32.4 compared to the reference strain *V. campbellii* BAA-1116.

## Discussion

Luminescent vibriosis is a major challenge in shrimp hatcheries^2^. Several earlier reports suggested luminescent bacterium *V. harveyi* as the causative agent. However, recent reports of misidentification between *V. harveyi* and closely related *V. campbellii* demand a reexamination of the causal association for luminescent vibriosis and the virulence factors determining the outcome of infection. To address the knowledge gap, we characterized 29 luminescent *Vibrio* isolates, 76% represented shrimp hatcheries, using 16S rRNA, *rpoD* and *toxR*. Based on the critical amino acid substitution at 123rd and 150th positions of virulence regulator ToxR*,* the quantified virulence markers were clustered to delineate the mechanism behind pathogenic and non-pathogenic isolates.

In the present study, routine laboratory identification methods such as phenotypic and biochemical characteristics identified all the isolates as *V. harveyi.* The 16S rRNA, a universal species delineating marker, produced a polyphyletic tree and failed to correctly identify the species. However, multiplex PCR based on haemolysin gene and phylogenetic analysis of *rpoD* and *toxR* confirmed all the isolates as *V. campbellii*. It is interesting to note that a few of the luminescent isolates used in the present study (LB1 and LB10) were earlier identified as *V. harveyi* (reported as VH1 and VH10) applying phenotypic and biochemical methods^4^. Similar to the present findings, 26 out of 28 earlier reported *V. harveyi*, were *V. campbellii*^28^*.* Likewise, *V. harveyi* ATCC BAA-1116, a strain widely used as a model for quorum sensing studies, has been reclassified as *V. campbellii* BAA-1116^16^. The authors also documented that 28% of *V. harveyi* isolates are misidentified. Based on the results from the present study and earlier reports, it can be concluded that *V. campb*e*llii* has remained a highly under-reported pathogen due to misidentification as *V. harveyi*.

The average nucleotide identity (ANI) of 16S rRNA gene sequence is considered as the gold standard for species delineation. A 97% ANI of 16S rRNA corresponding to 70% DNA-DNA hybridization has been widely used as the threshold to consider a member of the same species^9^. Recently, based upon the comparative analysis of 6787 prokaryotic genomes, the identity threshold for species demarcation by 16S rRNA has been raised to 98.65%^31^. The luminescent bacterial isolates in the present study showed an average of 99.87%, 99.30, and 98.99% identity with type strain *V. campbellii* CAIM519T, *V. owensii* CAIM 1854 T and *V. harveyi* ATCC 14,126, respectively (Supplementary Table S2). This is far beyond the limit for species delineation. Earlier, 97.6–99.9% identity in 16S rRNA within Harveyi clade was reported and was found inadequate to differentiate core members of Harveyi clade^7^. Thus, the present findings are in accordance with the earlier reports suggesting a lack of ability of 16S rRNA in differentiating closely related species within the Harveyi clade such as *V. harveyi* and *V. campbellii*.

Proteases such as serine proteases, metalloproteases, and cysteine proteases are the major virulence factors in several *Vibrio* spp such as *V. harveyi, V. alginolyticus, V. parahaemolyticus, V. anguillarum*^32^. The luminescent bacterium *V. harveyi* is known to produce cysteine protease^21^, serine protease^33^, and metalloprotease^34^ in strain-dependent manner. In the present study, metalloprotease was found to be the major protease of *V. campbellii* as it was completely inhibited by 1,10-phenanthroline and partially by EDTA (Table 2). A lower level of protease activity recorded in the least pathogenic groups AP and PP suggests its role in the pathogenicity. This is in consonance with the earlier study, which reported the protease role in the pathogenicity of *V. harveyi* in causing mortality to *P. monodon*^21^. However, the AQ and PQ groups did not differ significantly in the pathogenicity (Figure 5H) despite having a significant difference in the protease activity (Figure 5D). This indicates that although protease is a critical factor in pathogen virulence, the whole gamut of pathogenicity is not completely dependent upon it. Virulence of a pathogen is a multi-factorial phenomenon and decided by the interplay of several factors such as quorum sensing, colonization factors, secreted toxins, pir (photorabdus insect-related) toxin, RtxA (repeat in toxin), secretion systems, virulence regulators, etc^35–38^. Although pir toxin among *Vibrio* spp has emerged as major virulence determinants^38^, none of the isolates in the present study were found to carry pir toxin (Figure 4). Recently, we analysed the genome of *V. campbellii* LB102 and documented the presence of several virulence factors such as YopT (effector toxin of Type III secretion system), VgrG-3 (effector toxin of type VI secretion system), Rtx, and presence of Type I secretion system (T1SS), T2SS, T3SS and T6SS^17^. A comparative genomics analysis of pathogenic and non-pathogenic strains will be able to shed more light in the virulence of *V. campbellii.*

The transmembrane regulatory protein, ToxR is a major virulence regulator in human pathogens *V. cholerae* and *V. parahaemolyticus*^26,39^. To understand the possible role of ToxR in *V. campbellii,* we deduced the amino acid sequences between 74 to 250 amino acids (Figure 2). Although, initial 73 amino acids were not included in the study, the analysis of ToxR sequences of *V. campbellii* from NCBI database suggests the highly conserved nature of this region across the strains (personal observation). In this study, we analysed 29 isolates from our lab and eight strains from NCBI database for the ToxR protein, and the isolates were classified into four variants based upon critical amino acid substitution at 123rd and 150th position. The data from the present study suggests that ToxR had significant influence over the pathogenicity of *V. campbellii* as AQ variant had significantly higher pathogenicity than P150 variants AP and PP (Figure 5H and 8A). This could be attributed to deleterious Q150P substitution in AP and PP variants which has almost abolished the protease activity (Figure 5D). Apart from pathogenicity and protease production, variants of ToxR also significantly differed in quorum sensing signal autoinducer-2 (Figure 5C) and biofilm formation abilities (Figure 5E). Detailed functional analysis of ToxR protein has been carried out in *V. cholerae* where it controls the expression of more than 17 virulence genes, including *ctxAB* (cholera toxin) and *tcpA* (toxin-coregulated pilus, a colonization factor)^26^. In *V. parahaemolyticus* it has been reported to regulate the expression of type six secretion system 1 (T6SS1) by coordinating with quorum sensing regulators AphA and OpaR^39^. Further, syntenic organization of *toxS-toxR-htpG* operon in *V. campbellii* was similar to *V. cholerae* and *V. parahaemolyticus*. This strongly suggests the regulatory role of ToxR in the virulence pathway of *V. campbellii*. However, it is important to note that within a particular ToxR variant, a significant difference in pathogencity was observed among the strains (Figure 8B). This was specifically more pronounced in PQ variant where the CoxPH value ranged between 3 to 24.2. The large variation in mortality within PQ variant of ToxR suggests the involvement of other virulence factors and need further investigation.

Quorum sensing is a major virulence regulator in Harveyi clade spp^23^. It has been reported that quorum sensing downregulates most of the virulence markers such as T3SS^25^, chitinase^40^ and upregulates the metalloprotease expression in *V. harveyi*^25^. However, in the present study, the protease activity of *V. campbellii* had a strong negative correlation with quorum sensing autoinducer-2 signal. Regulation of genes by quorum sensing is population-dependent and differs from log to stationary phase of culture^23^. In the present study, genomic analysis of the strain *V. campbellii* LB102 revealed the presence of three genes for M4 zinc metalloprotease and a gene for M9 collagenase (Table 3). This is in accordance with an earlier report that suggested the presence of three genes for M4 metalloprotease and 14 other metalloproteases in *V. corallilyticus*^41^. The metalloproteases of M4 family such as EmpA of *V. anguillarum*^42^, VcpA of *V. coralliilyticus*^41^, VvpE of *V. vulnificus*^43^ have been characterized for pathogenic significance. Due to presence of large number of metalloproteases within *V. campbellii* genome, we speculate that different genes of metalloproteases may be upregulated or downregulated depending upon the growth phases of the pathogens. As protease production and quorum sensing signal AI-2 in four variants of ToxR changed with amino acid substitution, indicate that these factors are interlinked. We further hypothesize that the critical amino acid substitution in ToxR at 150th position (Q150P) could have disabled its regulatory function and subsequently resulted in many fold increase in the level of AI-2 signal which triggered a powerful metalloprotease inhibition. Further study is warranted to explore the mechanism behind the interplay of ToxR, quorum sensing and protease activity.

The survival analysis and Cox PH regression model were applied to understand the pathogenicity of *V. campbellii*. The Cox PH model take the consideration of total number of mortaltiy and the time-point at which mortality happened, hence become ideally suitable for analyzing relative pathogenicity of bacterial strains. In the present study, *V. campbellii* BAA-1116 had the lowest level of pathogenicity. Therefore, it was used as reference to estimate the relative pathogenicity (hazard ratio) of other strains. The study indicated that 72.4% of isolates had a significantly higher (p < 0.05) level of pathogenicity than *V. campbellii* BAA-1116. Even though the majority of the isolates were collected from the *P. monodon* shrimp hatcheries and farms (27 out of 29 isolates), these isolates were found to be highly pathogenic for *P. vannamei* and were not species-dependent. Bioluminescence is one of the most remarkable features for many of *Vibrio* species such as *V. campbellii, V. harveyi, V. fischeri, V. logie, V. salmonicida, V. orientalis, V. splendidus,* etc^44,45^. In the present study, a significant variation was observed in the quantum of luminescence production as reported earlier^13,45,46^. However, no significant correlation was observed between bioluminescence and pathogenicity, suggesting pathogenicity independent from the bioluminescent behavior of the pathogens.

In conclusion, the results from the present study suggest that *V. campbellii* is a predominant pathogen in Indian shrimp hatcheries, which has been largely mistaken in the past as *V. harveyi*. The comparative analysis among variants of ToxR revealed a highly significant variation in virulence factors and pathogenicity. The study found that the presence of glutamine at 150th position (Q150) in ToxR is crucial for the pathogenicity of *V. campbellii*. The protease analysis revealed its metalloprotease nature which appears to be under the regulatory control of ToxR and quorum sensing. A further study using site-directed mutagenesis at 123rd and 150th position of ToxR is worth investigating for deeper understanding on the virulence regulatory mechanism of *V. campbellii*.

## Methods

### Bacterial isolates and growth condition

Twenty-nine isolates of luminescent *Vibrio* isolated during 2006–2018 from penaeid shrimp hatcheries and grow-out farms in Tamil Nadu (11.1271° N, 78.6569° E) and Andhra Pradesh (15.9129° N, 79.7400° E), India, were used for the study (Table 1). The selection criteria aimed to ensure the larger diversity, such as (a) diseased hatchery and healthy farm (b) low vs. high luminescence (c) geographical origin (d) isolation period of more than ten years. Additionally, *V. campbellii* BAA1116 wild type (BB120) was used as a reference strain. For quorum-sensing signal quantification, the mutant strains of *V. campbellii* BAA1116 (BB170 and JMH612) were used. All the isolates were maintained as 25% glycerol stock at − 80 °C at Aquatic Animal Health and Environmental Division, ICAR-CIBA, Chennai, Tamil Nadu. Before the start of the experiment, the isolates were revived on Zobell Marine Agar (ZMA), tryptic soy agar (TSA) with 1.5% sodium chloride, and TCBS agar (HiMedia, Mumbai, India).

### Phenotypic characterization of *Vibrio* isolates

The phenotypic characterization of all the bacterial isolates was carried out as per standard methods^47^. In brief, the selected isolates were tested for oxidase test, utilization of amino acids (arginine hydrolase, lysine decarboxylase, and ornithine decarboxylase), carbohydrate fermentation tests (glucose, sucrose, mannitol, lactose, arabinose, cellobiose, galactose, sorbitol, and mannose), salt tolerance tests (growth at 0, 3, 6, 8 and 10% salt in peptone water), indole production, methyl red, Voges-Proskauer (VP) test, citrate utilization, nitrate production, and gelatinase tests. The maximum likelihood phylogenetic tree using *toxR* gene was visualized using iTOL v4 (Interactive Tree of Life) with an additional panel representing biochemical characteristics among *Vibrio* isolates^48^ (Supplementary Figure S1).

### Extraction of genomic DNA

Genomic DNA was extracted according to earlier described method^49^ with minor modifications. Briefly, 6 h old bacterial culture was pelleted and re-suspended in 400 µl Gram-negative bacterial lysis buffer (40 mM Tris–acetate pH 7.8, 20 mM sodium-acetate, 1 mM EDTA, 1% SDS). The viscous solution was incubated at 65 °C for 20 min and subsequently treated with RNase at 37 °C for 20 min. To remove proteins and cell debris, 132 µl of 5 M NaCl solution was added, mixed, and centrifuged at 15,000×*g* for 10 min at 4 °C. The clear supernatant was mixed with chloroform (1:1 v/v) and centrifuged at 15,000×*g* for 10 min at 4 °C. The upper aqueous layer was collected and mixed with 100% ethanol (1:1 v/v), and the DNA was precipitated by centrifugation at 15,000×*g* for 10 min at 4 °C. The pellet was washed twice with 70% ethanol, air-dried, and dissolved in 100 µl nuclease-free water (NFW). The DNA concentration was checked in Nanophotometer (Implen, Germany) and diluted to 50 ng µl^−1^ concentration for further study.

### Molecular identification of *Vibrio* isolates

#### Identification based on multiplex PCR of haemolysin gene

Species-specific multiplex PCR was carried out targeting the haemolysin (*hly*) gene for the identification of *V. harveyi* and *V. campbellii* as per the earlier described method^50^. The primers used for species-specific multiplex PCR study is presented in Supplementary Table S1.

#### Identification based on 16S rRNA, rpoD and toxR gene

Partial sequencing of the 16S rRNA, RNA polymerase sigma factor (*rpoD*) and transmembrane virulence regulator *toxR* was carried out for 29 test isolates using earlier reported primers^7,51^. The forward and reverse sequences were assembled in BioEdit^52^ and homology search was performed using BLASTN (http://www.ncbi.nih.gov/BLAST). The highest scoring hit and lowest *E* value were taken as possible matches^53^. To find the level of identity with reference strains, a local database was created using the 16S rRNA, *rpoD,* and *toxR* sequences of type strain *V. campbellii* CAIM 519T, *V. owensii* CAIM 1854, and *V. harveyi* ATCC 14126. The local BLAST was performed using default parameters except for *toxR* where word length was reduced to 7.

The gene sequences of 16S rRNA, *rpoD,* and *toxR* genes were aligned with sequences from other closely related *Vibrio* spp. by ClustalW, and phylogenetic tree was constructed using individual and concatenated gene sequences by maximum likelihood method (ML) applying MEGA X software^54^. The tree was rooted using *V. cholerae* N16961 as an outgroup. The robustness of each topology was checked by 1000 bootstrap replications. The partial 16S rRNA, *rpoD,* and *toxR* sequences of 29 isolates were deposited in the GenBank database under accession number MW425296-MW425324 and MW428977-MW429034.

### Characterization of *ToxR* protein

The nucleotide sequence of *toxR* of *V. campbellii* isolates was deduced into amino acid sequence by ExPASY translate tool (https://web.expasy.org/translate/). The deduced amino acid sequences of 29 Indian isolates and eight strains collected from the NCBI database were aligned by MUSCLE^55^ in the region between 74 to 250 amino acid and was demonstrated by the Jalview version 1.6^56^. To understand the syntenic organization of toxR gene loci, the protein sequences of ToxR and neighbouring proteins of *V. cholerae* N16961 were downloaded from NCBI database. The gene synteny among *V. cholerae, V. parahaemolyticus,* and *V. campbellii* was derived by the program simpleSynteny using TBLASTN with evalue 0.001 and minimum query coverage of 50%^57^.

The deduced amino acid sequence of virulence regulator ToxR suggested two critical substitutions among the isolates at 123rd (proline with alanine) and 150th position (glutamine with proline). Based upon these substitutions, isolates were grouped into four variants; A123Q150 (AQ), P123Q150 (PQ), A123P150 (AP), and P123P150 (PP). These ToxR variants were comparatively analysed for virulence markers and pathogenicity study in *P. vannamei*.

### Detection of virulence markers

The primers used for the characterization of virulence genes are presented in Supplementary Table S1. The primers for quorum sensing genes such as *luxLM* (harveyi autoinducer 1), *luxS* (Autoinducer 2), and *cqsA* (cholera autoinducer) were designed using primer3 software (https://Frodo.wi.mit.edu/primer3) based on the consensus gene sequence available in GenBank. The virulence markers such as *luxR, toxR, vhpA* (metalloprotease), *SePro* (serine protease), *chiA* (chitinase), *hly* (haemolysin), *flaA* (flagellin), *lafA* (lateral flagella) were screened as per earlier described methods^20,58^. Further, the virulence genes of well-established pathogenic *Vibrio* species such as cholera toxin (*ctx*) of *V. cholerae*^59^, thermostable direct hemolysin (*tdh*) and tdh-related haemolysin (*trh*) of zoonotic *V. parahaemolyticus*^60^, photorabdus insect-related toxin (*pirAB*) of AHPND strain of *V. parahaemolyticus*^61^ were also screened.

The PCR reaction was performed in 25 µl volume containing 12.5 µl Taq DNA polymerase red dye master mix (2 ×) (Ampliqon, Denmark), 1 µl forward primer (10 µM), 1 µl reverse primer (10 µM), 1 µl template DNA (50 ng) and 9.5 µl NFW. The amplification reaction was carried out under the following conditions: initial denaturation at 95 °C for 5 min followed by 35 cycles of denaturation at 95 °C for 1 min, annealing at 54 °C for 30 s (*luxR, toxR,* SePro, *vhpA*), 55 °C for 30 s (*pirAB*), 55 °C for 60 s (*tdh, trh*), 60 °C for 30 s (*vhh, luxLM, luxS, cqsA, flaA, lafA*), 60 °C for 60 s (*ctxA*), 62 °C for 30 s (*chiA, cqsA*) and extension at 72 °C for 1 min and final extension at 72 °C for 10 min. The amplified PCR products were loaded on 1.5% (w/v) agarose gel and resolved in 1 × TAE buffer. The gel was visualized and photographed under a gel documentation system (Bio-Rad, USA).

The maximum likelihood phylogenetic tree was visualized using iTOL v4 (Interactive Tree of Life) with an additional panel representing the distribution of virulence markers among isolates^48^ (Figure 4).

### Quantification of virulence markers

#### Bioluminescence assay

Bioluminescence analysis was carried out using Luria Bertani (LB) broth as per standard protocol^19^. In brief, *Vibrio* isolates were cultured in 10 ml LB broth in an orbital shaker (30 °C, 120 rpm). A 200 µl bacterial culture (in triplicate) was transferred in white opaque optiplate 96 (Perkin Elmer) at 6 and 12 h culture period. After keeping the plates in darkness for 10 min, the luminescence was measured (Tecon, Switzerland). The results were recorded as fold change of luminescence in samples over negative control (LB uninoculated medium).

#### Quorum sensing signal

For the quantification of quorum sensing signals harveyi autoinducer 1 (HAI-1) and autoinducer 2 (AI-2), *Vibrio* isolates were cultured in 10 mL LB broth using 0.5% inoculum. The bacterial culture was incubated at 30 °C, 120 rpm, and the sample was collected at 6 h and 12 h (OD_600_ = 1.8 to 2.0). The sample was centrifuged at 6000×*g* for 15 min at 4 °C. The culture supernatant was passed through a 0.45 µ syringe filter. This served as cell-free culture supernatant and was stored as an aliquot at –80 °C till further use.

The quantification of HAI-1 and AI-2 was done using the quorum-sensing sensor strain *V. campbellii* JMH612 (received courtesy Dr. Bassler) and *V. campbellii* BB170 (ATCC), respectively by earlier described methods^62^. Briefly, a single colony of sensor strain JMH612 and BB170 was transferred to 10 ml autoinducer bioassay medium^62^. The culture was incubated overnight at 28 °C, 100 rpm, till OD_600_ reached 1.0. A 10 μl sensor strain culture was transferred to 50 mL fresh autoinducer bioassay (AB) medium (5000-fold dilution). A 180 μl of diluted sensor strain was incubated with 20 μl cell-free culture supernatant of *Vibrio* isolates in a white opaque microplate (OptiPlate 96; PerkinElmer). The BB120, wild strain of *V. campbellii* BAA 1116, cell-free culture supernatant was used as the positive control. A 20 µL sterile LB medium in place of culture supernatant was used as the negative control. All the samples were run in triplicate. The luminescence was measured after incubating the plate for 3 h at 30 °C, 100 rpm. The autoinducer was quantified in terms of fold change in luminescence over the negative control using the following formula

$${\text{Autoinducer}}\;{\text{signal}}\;\left( {{\text{HAI{-}1}}/{\text{or}}\;{\text{AI{-}}}2} \right) = \frac{{{\text{Luminescence}}\;{\text{produced}}\;{\text{by}}\;{\text{sensor}}\;{\text{strain}}\;{\text{in}}\;{\text{the}}\;{\text{presence}}\;{\text{of}}\;{\text{cell}}-{\text{free}}\;{\text{culture}}\;{\text{supernatant}}\;of\;Vibrio\;{\text{isolates}}}}{{{\text{Luminescence}}\;{\text{produced}}\;{\text{by}}\;{\text{sensor}}\;{\text{strain}}\;{\text{with}}\;{\text{sterile}}\;{\text{LB}}\;{\text{medium}}}}$$

#### Protease test

The protease activity in the *Vibrio* supernatant was estimated by azocasein assay^63^. The main principle behind the azocasein assay is the separation of the azo-molecule from the casein protein during azocasein degradation by bacterial extracellular proteases. Briefly, 100 μl of azocasein (5 mg/ml) in 100 mM Tris (pH 8.0) was incubated with 100 μl of overnight grown cell culture supernatant and 50 μl of 100 mM Tris (pH 8.0) for 1 h at 37 °C. The reaction was stopped by the addition of 400 μl of 10% trichloroacetic acid. After centrifugation, the supernatant was transferred to 700 μl of 525 mM NaOH, and the optical density was determined at 442 nm (OD442). One azocasein unit was defined as the amount of enzyme producing an increase of 0.01 OD units per h.

#### Chitinase test

The chitinase produced by the luminescent *Vibrio* isolates was quantified using the method described by Reyes‐Ramírez^64^. In brief, 1 ml of enzyme sample, 1 ml of 0.2 M sodium phosphate buffer (pH 7), and 5 mg of chitin-azure were mixed and incubated at 37 °C for 3 h at 150 rpm. The tubes were centrifuged at 5000×*g* for 10 min, and the absorbance of the supernatants was read at 560 nm. One unit of chitinase was defined as the amount of enzyme that produced an increase of 0.01 units of absorbance.

#### Biofilm assay

Biofilm was quantified using 1 ml LB medium in borosilicate glass tubes (10 × 75 mm) and was inoculated with 10 µl of diluted (1:100) 3 h old culture (OD_600_ = 1)^65^. The culture was incubated at 30 °C for 18 h. After a mild rinse with distilled water, to detach the unattached bacterium over the glass surface, the tubes were stained with crystal violet (0.1%) for 5 min. After mildly washing the tubes with distilled water, the biofilm-associated crystal violet was resuspended in ethanol, and the OD was measured at 570 nm.

#### Motility assay

A motility test was carried out on Luria Bertani (LB) agar plates with 0.4% Bactoagar^58^. Bacterial colonies were stabbed into motility agar plates with a nichrome inoculation needle and incubated at 30 °C for 24 h. The diameter of each swarm was measured post 24 h incubation.

### Protease characterization

To find the nature of protease, the enzyme inhibition assay was carried out using the protease inhibitor such as ethylenediamine tetraacetic acid (EDTA; Sigma) and 1,10-phenanthroline (Sigma) for metalloprotease, phenyl-methanesulfonyl fluoride (PMSF) and benzamidine (Sigma) for serine protease, and p-hydroxymercuribenzoate (PHMB; Sigma) for cysteine protease. The final concentration of all the protease inhibitors was 1 mM except for EDTA, which was evaluated at 10 mM. The reaction was carried out as similar to protease except that the buffer volume of 50 μl was adjusted with the volume of inhibitor.

One of the respresentative isolate, *V. campbellii* LB102 genome was analysed for presence of metalloproteases with pathogenic significance. For this purpose, EmpA of *V. anguillarum* and collagenase of *V. alginolyticus* was downloaded from NCBI database and PBLAST analysis was carried out against protein sequences of *V. campbellii* LB102. A minimum 50% coverage and 30% identity at protein level was taken for presence of gene. Their extracellular nature was futher confirmed by PSORTb analysis^66^.

### Pathogenicity testing in *Penaeus vannamei*

White leg shrimp, *P. vannamei* juveniles reared at Muthukadu Experimental Station of ICAR-Central Institute of Brackishwater Aquaculture (ICAR-CIBA), Chennai, India were used for challenge study. The *P. vannamei* juveniles (4.2 ± 0.2 g) were injected intramuscularly with 50 µL of the bacterial inoculum (5 × 10^4^ CFU/shrimp) between 2nd and 3rd abdominal segment using tuberculin syringe (U-40, 31 gauze). The challenge dose was optimized by intramuscularly injecting LB3 (less pathogenic) and LB10 (more pathogenic) by doses between 10^3^ and 10^7^ CFU/shrimp (data not presented). The one LD_50_ of lower pathogenic isolate LB3 (~ 5 × 10^4^ CFU/shrimp) was used as the challenge dose for all the isolates. Briefly, 30 bacterial isolates (29 LB isolates and BAA-1116 as reference strain) were incubated at 30 °C for 18 h on fresh Zobell marine agar (HiMedia, Mumbai, India), and colonies were suspended in normal saline solution (NSS, 0.9% NaCl). After washing, the pellet was re-suspended in NSS and set the OD600 at 1.0 (~ 10^8^ CFU/ml) using UV–VIS spectrophotometer. Finally, the culture was diluted 100 times in NSS, and 50 µL of diluted culture was injected into each shrimp, providing a challenge dose of 5 × 10^4^ CFU/shrimp. The challenge trial was conducted in 100 L FRP tanks, with eight shrimps/isolate resulting in 30 experimental tanks. To bring repeatability, the challenge trial was conducted twice in a similar manner with two different batches of *P. vannamei,* resulting in 16 shrimps/isolate. Mortality recorded within 2 h of injection was regarded as death due to injection shock and discarded from the study and replaced with the challenge to the fresh animal. Post injection, mortality was recorded at 6, 12, 18, 24, 48, 72, 96, and 120 h.

### Statistical analysis

One-way ANOVA was carried out to assess the differences in quantitative virulence factors among the four variants of ToxR; AQ, PQ, AP, and PP based upon amino acid at 123rd and 150th position. If the result was significant, the ANOVA was followed by Tukey post hoc comparison to assess differences among the allelic forms. The post hoc comparison was carried out by rstatix package while graphics were prepared using ggpubr package in R4.0.2.

The principal component analysis (PCA) was carried out to find the association between mortality pattern observed in *P. vannamei* with various virulence markers (mortality, protease, bioluminescence, AI1, AI2, biofilm, chitinase, motility). Before analysis, all the variables were auto-scaled to a unit variance allowing each variable to contribute equally to the PCA model. The number of principal components and the factors were selected based upon eigenvalues > 1.00. All the analysis was carried out using the statistical Factoextra and FactoMineR package in R4.0.2^67^.

The correlation matrix among the virulence factors was estimated using the corrplot. The hierarchial cluster analysis was conducted to evaluate the clustering pattern of quantitative virulence data. The Euclidean distances between all the quantitative variables were calculated, and the average linkage algorithm was used to build the dendrogram. The cluster was verified by computing the correlation coefficient between cophenetic distance and the original distance generated by dist function in R. The data was converted into a dendrogram coupled with heatmap using the ComplexHeatmap package in R.

Survival rates among different ToxR substitution groups were estimated by ANOVA, and Kaplan–Meier method^68^, and the survival curve was compared by applying a log-rank test. Cox proportional hazard regression analysis (CoxPH) was performed to assess the relative contribution of 29 *Vibrio* isolates, reference strain BAA-1116, and control group (NSS) in inflicting the mortality on *P. vannamei* juveniles^69^. As control did not produce mortality, hence relative pathogenicity was estimated against the reference strain BAA1116, which was found as least virulent among all isolates. All the analysis was carried out using the survival and surviminer package in R4.0.2^70^.

## Supplementary Information

Supplementary Information.
