# Supplementary material for: Delineating virulence of Vibrio campbellii: a predominant luminescent bacterial pathogen in Indian shrimp hatcheries
Source: Sci Rep. 2021 Aug 4;11:15831. doi: 10.1038/s41598-021-94961-4 (PMC8339124; doi:10.1038/s41598-021-94961-4)
Supplement: Supplementary file 1 — Supplementary Information. [file 41598_2021_94961_MOESM1_ESM.pdf]

Delineating virulence of *Vibrio campbellii*: A predominant luminescent bacterial pathogen in Indian shrimp hatcheries

Sujeet Kumar[1]*, Chandra Bhushan Kumar[1,2], Vidya Rajendran[1], Nishawlini Abishaw[1], P.S. Shyne Anand[1], S. Kannapan[1], Viswas K. Nagaleekar[3], K. K. Vijayan[1], S.V. Alavandi[1]

1. ICAR-Central Institute of Brackishwater Aquaculture, 75, Santhome High Road, Chennai 600028, India
2. ICAR - National Bureau of Fish Genetic Resources, Canal Ring Road, Dilkusha Marg, Lucknow 226002, India
3. ICAR -Indian Veterinary Research Institute, Izatnagar, Bareilly 243122, India

*** Corresponding author**

Sujeet Kumar, ICAR-Central Institute of Brackishwater Aquaculture,
75, Santhome High Road, MRC Nagar, Chennai, 600 028, India.
Email: sujeetmicrobiol@gmail.com

Supplementary Table S1. Primer sequence used for the identification and virulence characterization of *Vibrio* isolates

Gene	Gene product	Primer sequence	Amplicon (bp)	Reference
**Primer sequence for isolate identification and characterization**				
16S rRNA	16S rRNA subunit	fD1: CCGAATTCGTCGACAACAGAGTTTGATCCTGGCTCAG<br>rP2: CCCGGGATCCAAGCTTACGGCTACCTTGTTACGACTT	1500	1
rpoD	Factor σ70 RNA polymerase	70F: ACGACTGACCCGGTACGCATGTAYATGMGNGARATGGGNCANGT<br>70R: ATAGAAATAACCAGACGTAAGTTNGCY TCNACCATYTCYTTYT	780	2
toxR	Transmembrane regulatory protein	toxrF: GANCARGGNTTYGARGTNGAYGAYTC<br>toxrR: TTDKKTTGNCCNCYNGTVGCDATNAC	477	2
vhh	*Vibrio harveyi* hemolysin	F: GAGTTCGGTTTCTTTCAAG<br>R: TGTAGTTTTTCGCTAATTTC	454	3
Vch	*Vibrio campbellii* hemolysin	F: CTATTGGTGGAACGCAC<br>R: GTATTCTGTCCATACAAAC	328	3
**Primer sequence for screening of virulence genes**				
**luxM*	*Vibrio harveyi* Autoinducer 1	F: TTTACACCCTCAACGCTGTC<br>R: AACACTTCGCAAACGGCTTG	98	This study (VIBHAR_02765)
**luxS*	S-ribosylhomocysteine lyase	F: TCGTGTGGCTAAAACGATGC<br>R: AAGCCTGCGTACAAATGCTC	132	This study (VIBHAR_03484)

Gene	Gene product	Primer sequence	Amplicon (bp)	Reference
	(AI-2 synthesis)			
**cqsA*	Cholera autoinducer 1 synthesis	F: ATGGCGCGTGAGTTTGATTG<br>R: ACCAAGCCAGAACCATTTGG	83	This study (VIBHAR_ 06088)
luxR	Transcriptional activator for quorum-sensing	F: ATGGACTCAATTGCAAAGAG<br>R: TTAGTGATGTTCACGGTTGT	618	4
toxR$_{vh}$	Transmembrane regulatory protein	F: CGACAACCAAAATACGGAA<br>R: AGAGCAATTTGCTGAAGCTA	131	4
chiA	Chitinase	F: GGAAGATGGCGTGATTGACT<br>R: GGCATCAATTTCCCAAGAGA	232	4
flaA	Flagellin	F: CTGCGGGTCTTCAAATCTC<br>R: GTTAGTGGTCTCGTTCATTGC	128	5
lafA	Lateral flagella	F: AACACATCTGCAGCGAAAGG<br>R: TAGCGACATTGCCATGCTTG	144	5
vhpA	Metalloprotease	F: CTGAACGACGCCCATTATTT<br>R: CGCTGACACATCAAGGCTAA	201	4
SePro	Serine protease	F: TGCACGACCAGTTGCTTTAG<br>R: AAGTGGTCGTCAGCAAATCC	232	4
hly	Hemolysin	F: CTATTGGTGGAACGCAC<br>R: GTATTCTGTCCATACAAAC	328	3
ctxA	Cholera toxin	F: CGGGCAGATTCTAGACCTCCTG<br>R: CGATGATCTTGGAGCATTCCCAC	564	6
Tdh	Thermostable direct haemolysin	TdhD3; 5’- CCACTACCACTCTCATATGC-3′<br>TdhD5; 5’-GGTACTAAATGGCTGACATC-3′	251	7
Trh	TDH-related haemolysin gene	TrhR2; 5′-GGCTCAAAATGGTTAAGCG-3′<br>TrhR6; 5′-CATTTCCGCTCTCATATGC-3′	235	7

Gene	Gene product	Primer sequence	Amplicon (bp)	Reference
pirA/B	Photorabdus insect-related toxin	AP4-F1; ATGAGTAACAATATAAAACATGAAAC AP4-R1; ACGATTTCGACGTTCCCCAA	1269	8
pirA	Photorabdus insect-related toxin	TUMSAT-Vp3 F; GTGTTGCATAATTTTGTGCA TUMSAT-Vp3 R; TTGTACAGAAACCACGACTA	360	9

* Primers designed under this study. The accession number used for designing primer has been presented in parenthesis.

1. Weisburg, W. G., Barns, S. M., Pelletier, D. A. & Lane, D. J. 16S ribosomal DNA amplification for phylogenetic study. *J. Bacteriol.* **173**, 697–703 (1991).

2. Pascual, J., Macián, M. C., Arahal, D. R., Garay, E. & Pujalte, M. J. Multilocus sequence analysis of the central clade of the genus Vibrio by using the 16S rRNA, recA, pyrH, rpoD, gyrB, rctB and toxR genes. *Int. J. Syst. Evol. Microbiol.* **60**, 154–165 (2010).

3. Haldar, S. *et al.* Development of a haemolysin gene-based multiplex PCR for simultaneous detection of Vibrio campbellii, Vibrio harveyi and Vibrio parahaemolyticus. *Lett. Appl. Microbiol.* **50**, 146–152 (2010).

4. Ruwandeepika, H. A. D. *et al.* Presence of typical and atypical virulence genes in vibrio isolates belonging to the Harveyi clade. *J. Appl. Microbiol.* **109**, 888–899 (2010).

5. Yang, Q. & Defoirdt, T. Quorum sensing positively regulates flagellar motility in pathogenic V ibrio harveyi. *Environ. Microbiol.* **17**, 960–968 (2015).

6. Fields, P. I., Popovic, T., Wachsmuth, K. & Olsvik, Ø. Use of polymerase chain reaction for detection of toxigenic Vibrio cholerae O1 strains from the Latin American cholera epidemic. *J. Clin. Microbiol.* **30**, 2118–2121 (1992).

7. Tada, J. *et al.* Detection of the thermostable direct hemolysin gene (tdh) and the thermostable direct hemolysin-related hemolysin gene (trh) of Vibrio parahaemolyticus by polymerase chain reaction. *Mol. Cell. Probes* **6**, 477–487 (1992).

8. Dangtip, S. *et al.* AP4 method for two-tube nested PCR detection of AHPND isolates of Vibrio parahaemolyticus. *Aquac. Reports* **2**, 158–162 (2015).

9. Tinwongger, S. *et al.* Development of PCR diagnosis for shrimp acute hepatopancreatic necrosis disease (AHPND) strain of Vibrio parahaemolyticus. 魚病研究 **49**, 159–164 (2014).

Supplementary Table S2. Identification of *Vibrio* isolates based on BLAST analysis of 16S rRNA, *toxR* and *rpoD* genes

Gene	Bacterial strain	NCBI accession No.	*V. campbellii* CAIM519T			*V. owensii* CAIM 1854			*V. harveyi* ATCC 14126		
			Identity %	E value	Bit score	Identity %	E value	Bit score	Identity %	E value	Bit score
16S rRNA	LB1	MW425296	99.93	0	2584	99.36	0	2542	98.93	0	2503
16S rRNA	LB3	MW425297	99.86	0	2579	99.29	0	2536	99.00	0	2508
16S rRNA	LB10	MW425298	99.93	0	2584	99.36	0	2542	98.93	0	2503
16S rRNA	LB14	MW425299	99.86	0	2579	99.29	0	2536	99.00	0	2508
16S rRNA	LB16	MW425300	99.86	0	2579	99.29	0	2536	99.00	0	2508
16S rRNA	LB25	MW425301	99.93	0	2584	99.36	0	2542	98.93	0	2503
16S rRNA	LB27	MW425302	99.86	0	2579	99.29	0	2536	99.00	0	2508
16S rRNA	LB33	MW425303	99.86	0	2579	99.29	0	2536	99.00	0	2508
16S rRNA	LB37	MW425304	99.86	0	2579	99.29	0	2536	99.00	0	2508
16S rRNA	LB39	MW425305	99.93	0	2584	99.36	0	2542	98.93	0	2503
16S rRNA	LB67	MW425306	99.86	0	2579	99.29	0	2536	99.00	0	2508
16S rRNA	LB102	MW425307	99.93	0	2584	99.36	0	2542	98.93	0	2503
16S rRNA	LB131	MW425308	99.86	0	2579	99.29	0	2536	99.00	0	2508
16S rRNA	LB135	MW425309	99.93	0	2584	99.36	0	2542	98.93	0	2503
16S rRNA	LB157	MW425310	99.86	0	2579	99.29	0	2536	99.00	0	2508
16S rRNA	LB164	MW425311	99.86	0	2579	99.29	0	2536	99.00	0	2508
16S rRNA	LB171	MW425312	99.86	0	2579	99.29	0	2536	99.00	0	2508
16S rRNA	LB178	MW425313	99.86	0	2579	99.29	0	2536	99.00	0	2508
16S rRNA	LB186	MW425314	99.86	0	2579	99.29	0	2536	99.00	0	2508
16S rRNA	LB195	MW425315	99.86	0	2579	99.29	0	2536	99.00	0	2508
16S rRNA	LB198	MW425316	99.86	0	2579	99.29	0	2536	99.00	0	2508
16S rRNA	LB204	MW425317	99.86	0	2579	99.29	0	2536	99.00	0	2508
16S rRNA	LB210	MW425318	99.86	0	2579	99.29	0	2536	99.00	0	2508
16S rRNA	LB217	MW425319	99.86	0	2579	99.29	0	2536	99.00	0	2508
16S rRNA	LB235	MW425320	99.86	0	2579	99.29	0	2536	99.00	0	2508
16S rRNA	LB314	MW425321	99.86	0	2579	99.29	0	2536	99.00	0	2508

Gene	Bacterial strain	NCBI accession No.	*V. campbellii* CAIM519T			*V. owensii* CAIM 1854			*V. harveyi* ATCC 14126		
			Identity %	E value	Bit score	Identity %	E value	Bit score	Identity %	E value	Bit score
16S rRNA	LB503	MW425322	99.86	0	2579	99.29	0	2536	99.00	0	2508
16S rRNA	LB515	MW425323	99.86	0	2579	99.29	0	2536	99.00	0	2508
16S rRNA	LB516	MW425324	99.86	0	2579	99.29	0	2536	99.00	0	2508
toxR	LB1	MW429006	94.40	0	769	82.84	8.44E-138	475	74.81	9.45E-58	209
toxR	LB3	MW429007	94.00	0	758	82.87	8.44E-138	475	74.90	4.37E-61	220
toxR	LB10	MW429008	94.80	0	780	83.24	3.90E-141	486	75.15	9.39E-63	226
toxR	LB14	MW429009	94.60	0	774	82.65	3.93E-136	470	74.95	2.03E-59	215
toxR	LB16	MW429010	94.40	0	769	82.71	3.93E-136	470	74.95	4.37E-61	220
toxR	LB25	MW429011	94.40	0	769	82.84	8.44E-138	475	74.81	9.45E-58	209
toxR	LB27	MW429012	94.20	0	763	83.05	1.81E-139	481	74.95	2.03E-59	215
toxR	LB33	MW429013	94.80	0	780	82.84	8.44E-138	475	75.15	4.37E-61	220
toxR	LB37	MW429014	94.40	0	769	82.71	3.93E-136	470	74.95	4.37E-61	220
toxR	LB39	MW429015	94.80	0	780	83.24	3.90E-141	486	75.15	9.39E-63	226
toxR	LB67	MW429016	94.20	0	763	83.05	1.81E-139	481	74.95	2.03E-59	215
toxR	LB102	MW429017	94.80	0	780	82.84	8.44E-138	475	75.15	4.37E-61	220
toxR	LB131	MW429018	94.80	0	780	82.84	8.44E-138	475	75.15	4.37E-61	220
toxR	LB135	MW429019	94.80	0	780	83.24	3.90E-141	486	75.15	9.39E-63	226
toxR	LB157	MW429020	94.20	0	763	83.05	1.81E-139	481	74.95	2.03E-59	215
toxR	LB164	MW429021	94.60	0	774	82.65	3.93E-136	470	74.95	2.03E-59	215
toxR	LB171	MW429022	94.20	0	763	83.05	1.81E-139	481	74.95	2.03E-59	215
toxR	LB178	MW429023	94.20	0	763	83.05	1.81E-139	481	74.95	2.03E-59	215
toxR	LB186	MW429024	94.20	0	763	83.05	1.81E-139	481	74.95	2.03E-59	215
toxR	LB195	MW429025	94.80	0	780	83.24	3.90E-141	486	75.15	9.39E-63	226
toxR	LB198	MW429026	94.80	0	780	82.84	8.44E-138	475	75.15	4.37E-61	220
toxR	LB204	MW429027	94.80	0	780	82.84	8.44E-138	475	75.15	4.37E-61	220
toxR	LB210	MW429028	94.20	0	763	83.05	1.81E-139	481	74.95	2.03E-59	215
toxR	LB217	MW429029	94.20	0	763	83.05	1.81E-139	481	74.95	2.03E-59	215
toxR	LB235	MW429030	94.80	0	780	82.84	8.44E-138	475	75.15	4.37E-61	220

Gene	Bacterial strain	NCBI accession No.	*V. campbellii* CAIM519T			*V. owensii* CAIM 1854			*V. harveyi* ATCC 14126		
			Identity %	E value	Bit score	Identity %	E value	Bit score	Identity %	E value	Bit score
toxR	LB314	MW429031	94.80	0	780	82.84	8.44E-138	475	75.15	4.37E-61	220
toxR	LB503	MW429032	94.80	0	780	83.24	3.90E-141	486	75.15	9.39E-63	226
toxR	LB515	MW429033	94.20	0	763	82.53	1.83E-134	464	74.75	2.03E-59	215
toxR	LB516	MW429034	94.60	0	774	83.05	1.81E-139	481	74.56	4.40E-56	204
rpoD	LB1	MW428977	99.13	0	1452	96.04	0	1314	95.79	0	1303
rpoD	LB3	MW428978	99.13	0	1452	96.04	0	1314	95.79	0	1303
rpoD	LB10	MW428979	99.01	0	1447	95.91	0	1308	95.66	0	1297
rpoD	LB14	MW428980	99.63	0	1474	96.53	0	1336	96.16	0	1319
rpoD	LB16	MW428981	99.26	0	1458	96.16	0	1319	95.91	0	1308
rpoD	LB25	MW428982	99.26	0	1458	96.16	0	1319	95.91	0	1308
rpoD	LB27	MW428983	99.26	0	1458	96.16	0	1319	95.91	0	1308
rpoD	LB33	MW428984	99.26	0	1458	96.16	0	1319	95.91	0	1308
rpoD	LB37	MW428985	99.26	0	1458	96.16	0	1319	95.91	0	1308
rpoD	LB39	MW428986	99.26	0	1458	96.16	0	1319	95.91	0	1308
rpoD	LB67	MW428987	99.26	0	1458	96.16	0	1319	95.91	0	1308
rpoD	LB102	MW428988	99.26	0	1458	96.16	0	1319	95.91	0	1308
rpoD	LB131	MW428989	99.26	0	1458	96.16	0	1319	95.91	0	1308
rpoD	LB135	MW428990	99.13	0	1452	96.04	0	1314	95.79	0	1303
rpoD	LB157	MW428991	99.26	0	1458	96.16	0	1319	95.91	0	1308
rpoD	LB164	MW428992	99.26	0	1458	96.16	0	1319	95.91	0	1308
rpoD	LB171	MW428993	99.26	0	1458	96.16	0	1319	95.91	0	1308
rpoD	LB178	MW428994	99.26	0	1458	96.16	0	1319	95.91	0	1308
rpoD	LB186	MW428995	99.26	0	1458	96.16	0	1319	95.91	0	1308
rpoD	LB195	MW428996	99.26	0	1458	96.16	0	1319	95.91	0	1308
rpoD	LB198	MW428997	99.26	0	1458	96.16	0	1319	95.91	0	1308
rpoD	LB204	MW428998	99.26	0	1458	96.16	0	1319	95.91	0	1308
rpoD	LB210	MW428999	99.26	0	1458	96.16	0	1319	95.91	0	1308
rpoD	LB217	MW429000	99.26	0	1458	96.16	0	1319	95.91	0	1308

**Gene**	**Bacterial strain**	**NCBI accession No.**	***V. campbellii* CAIM519T**			***V. owensii* CAIM 1854**			***V. harveyi* ATCC 14126**		
			**Identity %**	**E value**	**Bit score**	**Identity %**	**E value**	**Bit score**	**Identity %**	**E value**	**Bit score**
rpoD	LB235	MW429001	99.26	0	1458	96.16	0	1319	95.91	0	1308
rpoD	LB314	MW429002	99.26	0	1458	96.16	0	1319	95.91	0	1308
rpoD	LB503	MW429003	99.01	0	1447	95.91	0	1308	95.79	0	1303
rpoD	LB515	MW429004	99.26	0	1458	96.16	0	1319	96.04	0	1314
rpoD	LB516	MW429005	99.26	0	1458	96.16	0	1319	95.91	0	1308

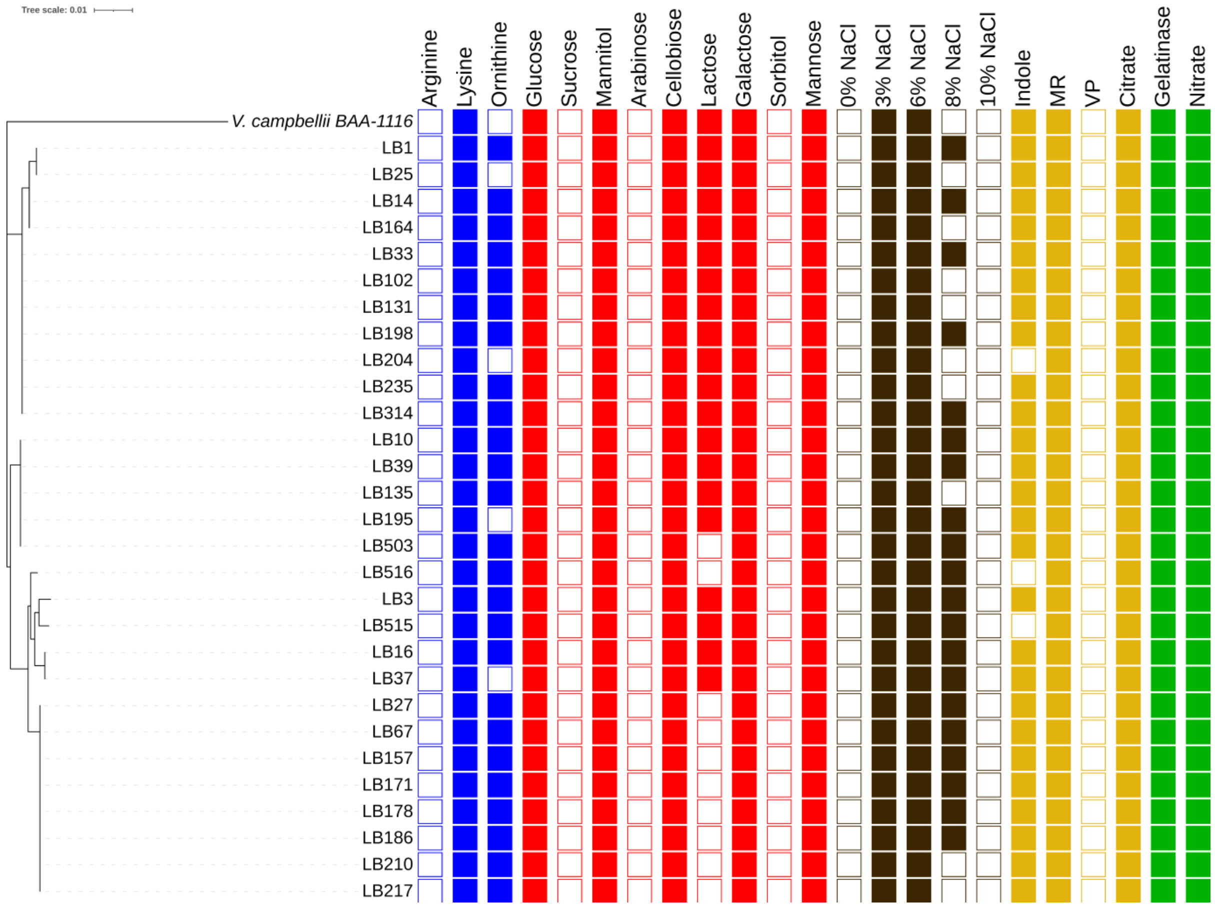

Supplementary Figure S1. Biochemical characteristics of luminescent bacterial isolates.

The phylogenetic tree was constructed using *toxR* nucleotide sequences applying maximum likelihood algorithm. The positive and negative biochemical tests were depicted by additional panel using iTOL v4 (Interactive Tree of Life). The blue, red, brown and green shaded box represents positive while white square box represents negative test. The MR and VP represent Methyl red and Voges-Praskauer test respectively.

Multiple exposure # 1

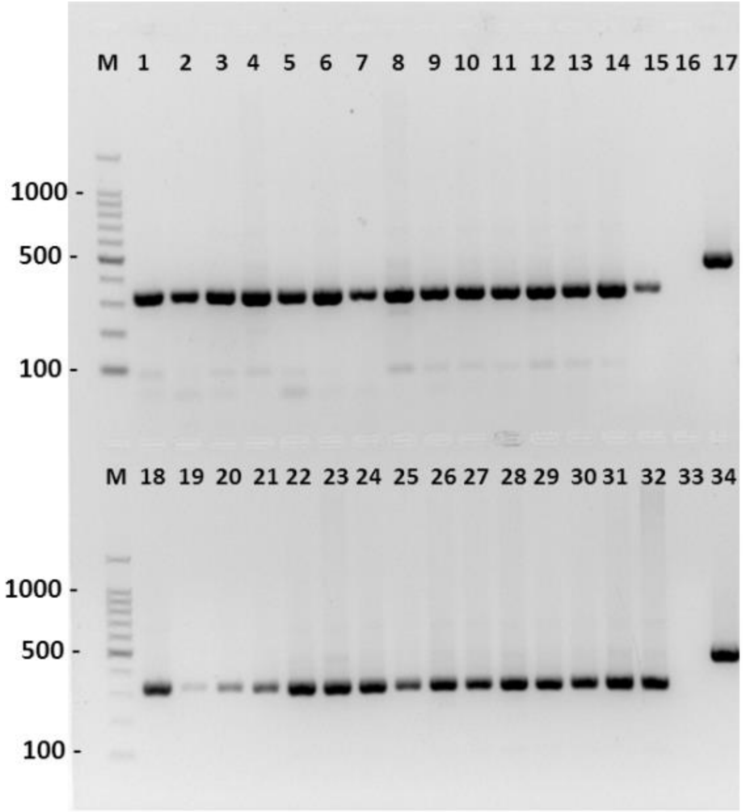

Multiple exposure # 2

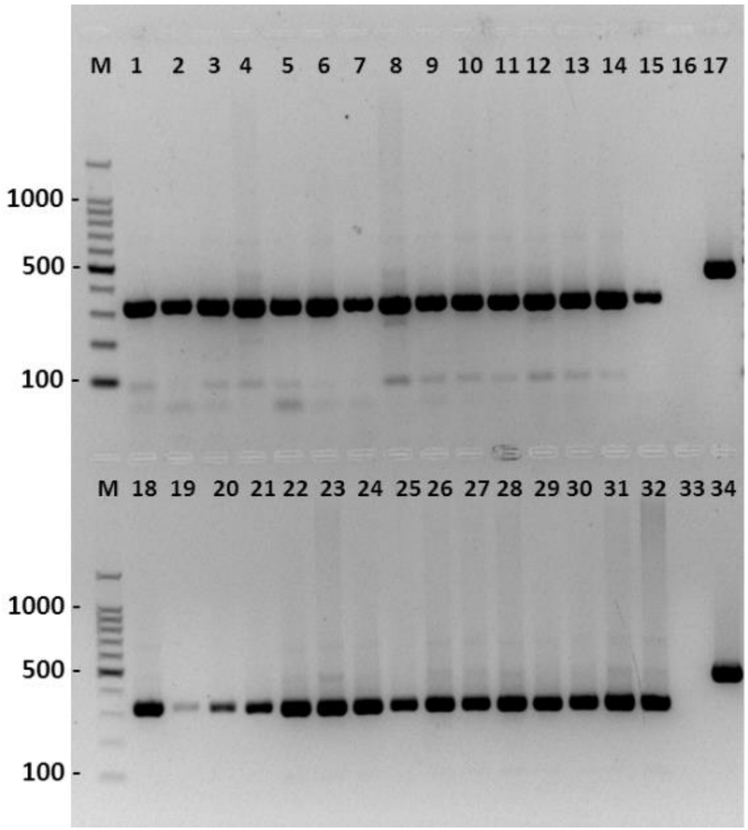

Multiple exposure # 3

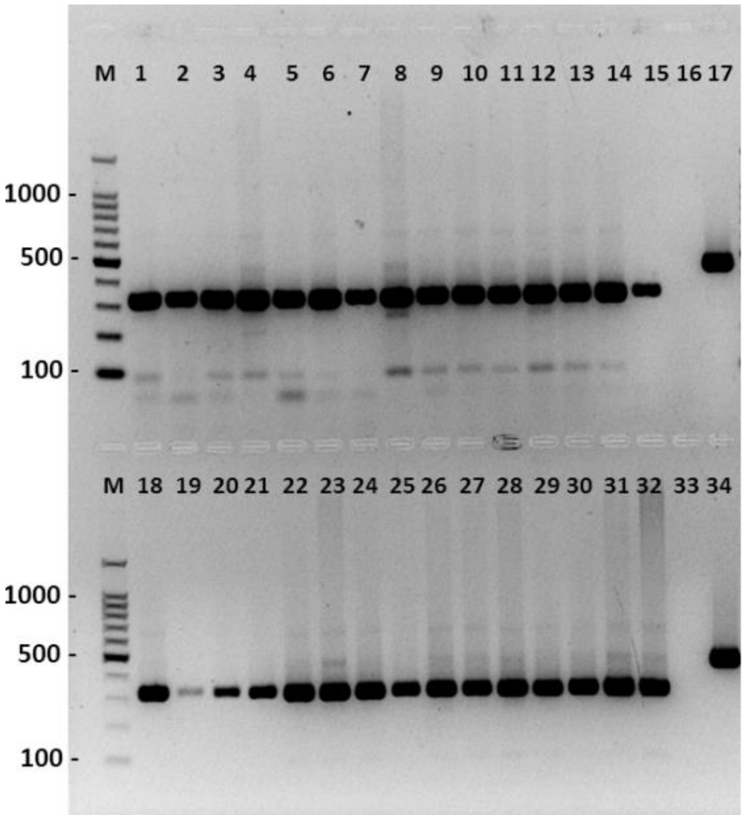

Supplementary Figure S2. Identification of luminescent bacterial isolates using hemolysin gene.

M: 100 bp DNA ladder (Takara); lane 1 to 15 and lane 18 to 31: 29 LB isolates in the series from LB1 to LB516; lane 32: reference strain *V. campbellii* BAA-1116; lane 16 and 33: Negative control; lane 17 and 34: *V. harveyi* LMG 4044

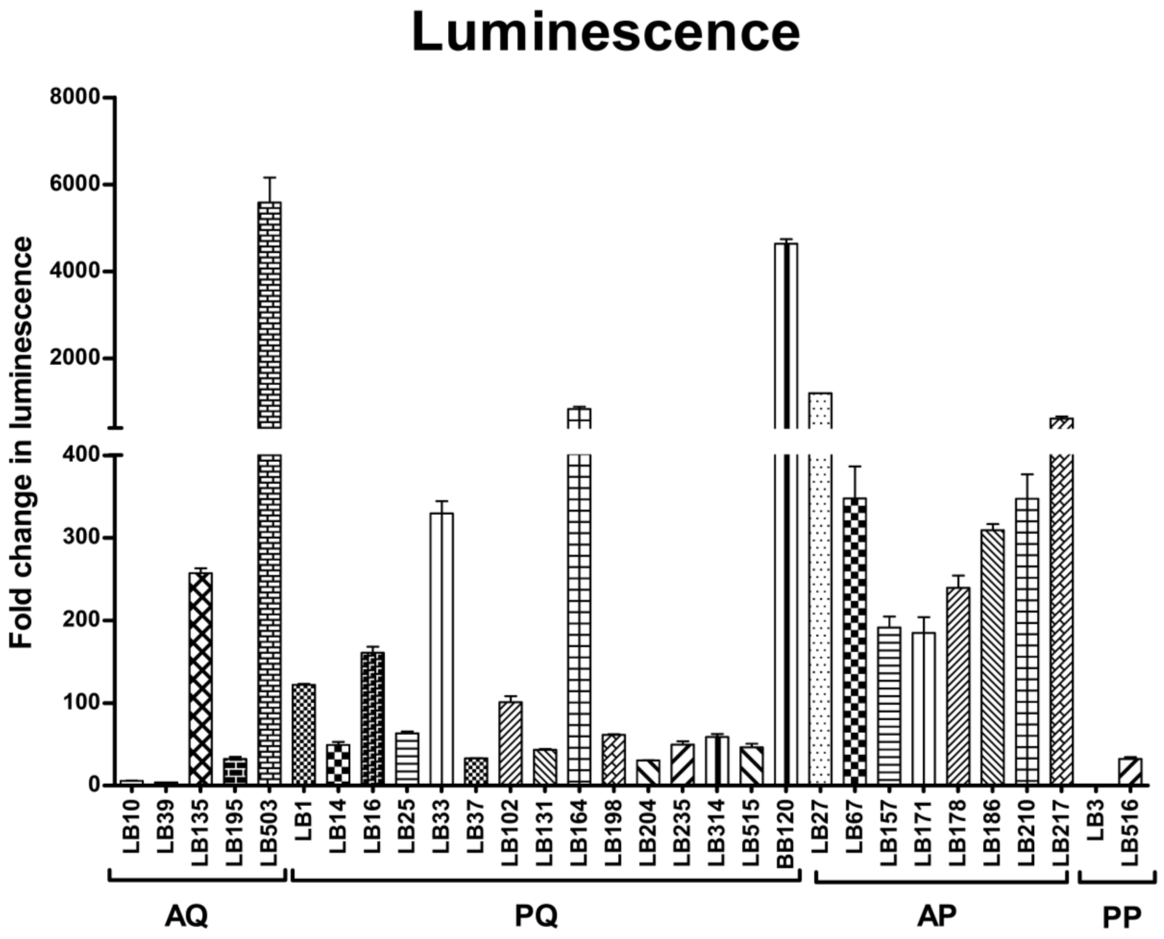

Supplementary Figure S3. Luminescence of *Vibrio campbellii* isolates.

The AQ (A123Q150), PQ (P123Q150), AP (A123P150) and PP (P123P150) represents four variants of ToxR based upon amino acid at 123rd and 150th position.
